# Do Fossil Fuel Subsidies Crowd Out Health Expenditure? A Country‐Level Longitudinal Analysis

**DOI:** 10.1002/hec.70074

**Published:** 2025-12-20

**Authors:** Judite Gonçalves, Eduardo Costa, Thomas Hone, Damini Singh, Paula Pereda, Anthony A. Laverty, Christopher Millett

**Affiliations:** ^1^ Public Health Policy Evaluation Unit Imperial College London London UK; ^2^ NOVA National School of Public Health Public Health Research Centre Comprehensive Health Research Center CHRC NOVA University Lisbon Lisbon Portugal; ^3^ CEGIST—Centro de Estudos de Gestão Instituto Superior Técnico Instituto Superior Técnico University of Lisbon Lisbon Portugal; ^4^ Instituto de Estudos para Políticas de Saúde (IEPS) São Paulo Brazil; ^5^ Economics and Social Sciences Area Indian Institute of Management Visakhapatnam Andhra Pradesh India; ^6^ Faculty of Economics University of São Paulo São Paulo Brazil; ^7^ Center for Research on Population and Health Faculty of Health Sciences American University of Beirut Beirut Lebanon

**Keywords:** country panel, fossil fuel subsidies, health expenditure, instrumental variable, oil price shock

## Abstract

Annually, countries allocate hundreds of millions of dollars to subsidize fossil fuels, often at the expense of public health and environmental sustainability. This undermines progress toward Sustainable Development Goals (SDG) 3 (Good Health and Well‐Being) and 13 (Climate Action). Despite this, the impact of fossil fuel subsidies (FFS) on social protection expenditure, including health, remains poorly quantified. This study aimed to determine whether FFS crowd out health expenditure globally, using panel data from 126 countries covering the period 2015–2019. An instrumental variable approach, relying on countries' exposure to international energy trade and fluctuations in crude oil price, was employed to capture exogenous variation in FFS and estimate a causal relationship. The analyses revealed that in 2019, 17 countries spent more than five percent of GDP on FFS, with FFS expenditure exceeding health expenditure in 15 of those countries. Specifically, a 1% increase in FFS per capita, driven by rising international oil prices and weighted by countries' exposure to international energy trade, led to a 0.05% (95% CI −0.08% to −0.02%) decrease in domestic health expenditure per capita. These findings underscore the detrimental impact of FFS on health expenditure, presenting another reason to eliminate FFS to achieve SDG3 in addition to avoiding further dangerous climate heating.

## 1 Introduction

Fossil fuel subsidies (FFS) have become a focal point in discussions about decarbonization of economies. In 2022, explicit FFS reached $1.3 trillion worldwide —a more than 100% increase from the previous year, largely driven by the Russian invasion of Ukraine and the subsequent energy crisis (IEA 2023; Black et al. 2023). Explicit FFS include a variety of support measures for both producers and consumers of fossil fuels (coal, natural gas, petroleum products, and electricity and heat generated from them) that lower retail prices below supply costs (implicit FFS are an estimation of the costs of air pollution and climate change, not considered here; Black et al. 2023). Abrupt changes in international energy prices can dramatically impact countries' FFS expenditure and consequently government finances.

Despite the scale of FFS, their impact on social protection expenditure, including health, remains poorly understood (Ebeke and Ngouana 2015). Peltovuori's study found that FFS in Kiribati equated to approximately 29% of public health expenditure during 2011–2014 (Peltovuori 2017). Other studies have highlighted the broader fiscal constraints imposed by FFS, suggesting that these subsidies often divert resources from critical sectors like health (Romanello et al. 2022; Gupta et al. 2015; Klaiber et al. 2023). For instance, when Indonesia significantly cut its gasoline and diesel subsidies in 2014, the government realized savings exceeding 10% of its total expenditure, which were partly reinvested in health (Pradiptyo et al. 2015). Similar reallocations have been observed in Ghana, Iran, and Morocco (Alers and Jones 2021; Crawford 2012; Merrill et al. 2016), where FFS reforms allowed for increased budget provisions for health and other social spending.

However, previous studies have been limited by their narrow focus on specific countries and their largely descriptive nature, which hinders the ability to draw causal inference between FFS and health expenditure. To fully understand this relationship on a global scale, more comprehensive analyses are required —particularly those that examine how health expenditure responds when fossil fuel market volatility and shocks cause sudden increases in public expenditure on FFS. Understanding the relationship between FFS and health expenditure may also inform whether cutting FFS might free up resources for health investments, a critical consideration for many countries struggling to finance expansions in health coverage (Zeng et al. 2020; Cylus et al. 2012). This issue is especially relevant now, as more countries are reforming their FFS regimes, such as Angola and Uzbekistan (World Bank 2023; IMF 2024).

This study starts by comprehensively analyzing associations between FFS, health expenditure, and the World Health Organization (WHO)'s Universal Health Coverage (UHC) Service Coverage Index across 126 countries in 2019. Then, it identifies the causal impact of FFS on health expenditure by employing panel data from those 126 countries over 2015–2019 and an instrumental variable that captures shifts in FFS from countries' exposure to fluctuations in international energy prices (i.e., energy imports interacted with crude oil prices).

## 2 Conceptual Background

**FIGURE 1:**
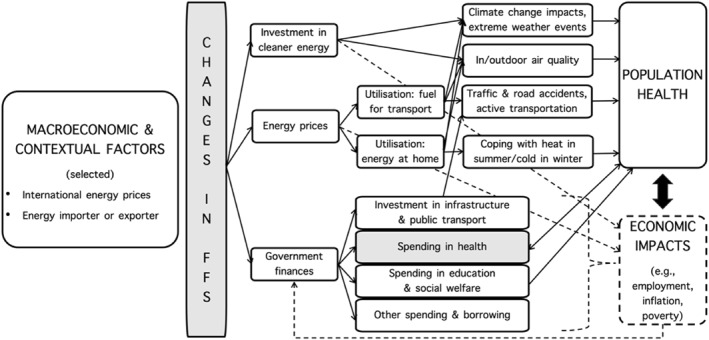
Conceptual framework linking FFS and health expenditure. *Source:* Own elaboration.

Variations in FFS impact three key variables that simultaneously relate to or impact health expenditure (Figure 1). First, FFS affect *population health* in multiple ways. Through incentivizing overconsumption of fossil fuels, subsidies contribute to increased transport‐related injuries and mortality as well as both indoor and outdoor air pollution (Black et al. 2023; Coady et al. 2017). Ambient air pollution from fossil fuel use generates an estimated 5.13 million excess deaths per year globally (Lelieveld et al. 2023). Klaiber et al. estimate health co‐benefits of removing FFS across 25 low‐ and middle‐income countries (LMICs) at 360,000 lives saved by 2035, through improved air quality alone (Klaiber et al. 2023). Associated climate change harms population health through increased heat‐related mortality and morbidity, extreme weather events, transmission of certain infectious diseases, and by increasing food insecurity (Romanello et al. 2022).

Second, FFS impact *economic activity* and have a range of distributional impacts (Mundaca 2017; Dennis 2016; del Granado et al. 2012; Ginn 2024; Jiang and Lin 2014; Li et al. 2017; Coady et al. 2015; Solarin 2021), which in turn may affect population health through the social determinants of health. For example, eliminating FFS could raise GDP by up to 0.65% in Malaysia (Li et al. 2017). Mundaca (2017) predicts that a 20 US$ cents increase in gasoline/diesel price per liter, through removal of subsidies, could increase the growth rate of GDP per capita by about 0.48%/0.30%. Distributional impacts are important, with Coady et al. (2015) summarizing evidence that the richest 20% of households captures, on average, more than six times more in fuel subsidies than the poorest 20%; however, subsidy withdrawal harms mostly the poor.

Third and most directly, FFS impact *government finances*, as their cost must be supported by government revenues (mainly taxes, also affected by changes in economic activity), borrowing, or cutting spending or investments in other areas. Coady et al. (2017) show that FFS depress long‐run growth and estimate that replacing subsidized energy prices with prices fully reflecting supply and environmental costs could raise government revenues by more than 10% globally (more than most governments collect from corporate income taxes).

## 3 Methods

### 3.1 Study Design and Data Sources

In this country‐level longitudinal study, we combined FFS data from the International Monetary Fund (IMF), health expenditure data from WHO, and other information from multiple sources described in the Appendix (Supporting Information S1: Table A1). Our initial panel dataset included 140 countries with FFS, health expenditure, and energy imports data (required to construct our instrumental variable) between 2015 (the first year FFS estimates are available) and 2019 (the last year some covariates are available). We excluded an additional 14 countries with zero FFS throughout the sample period, leaving a final panel covering 126 countries with 573 yearly observations (i.e., slightly unbalanced panel). Many excluded countries were small and island nations. The list of included countries is available in Appendix (Supporting Information S1: Table A2). For the descriptive analyses, we also obtained data on WHO's UHC Service Coverage Index, available from WHO's Global Health Observatory.

### 3.2 Fossil Fuel Subsidies

The IMF estimates FFS for almost all countries and defines explicit subsidies for a given fuel product as the difference between the supply cost per unit and the end‐user price, multiplied by the number of units consumed (i.e., price‐gap approach; Black et al. 2023; IMF 2013). The supply cost includes the price paid in the international energy market or the domestic production cost, as well as transportation, storage, and distribution costs. The end‐user price reflects measures to keep consumer prices artificially low, such as fixed consumer prices or rebates for energy purchases. Therefore, explicit FFS have a direct link to government budgets (e.g., rebates to consumers), or at least an indirect one (e.g., losses or reduced profits at state‐owned enterprises). Producer subsidies (e.g., favorable tax treatment for fossil fuel extraction) are included; however, the estimates do not separate between consumer and producer subsidies. It is possible to separate coal, natural gas, petroleum products, and electricity subsidies.

We obtained IMF estimates of explicit FFS in constant US dollars ($) and converted them to per capita amounts for the main analyses, or to percent of GDP for some descriptive analyses. We considered subsidies across all fossil fuels, as well as subsidies for petroleum products specifically in sensitivity analyses.

### 3.3 Health Expenditure

Three health expenditure variables were extracted from WHO's Global Health Expenditure Database: (1) government health expenditure, funded by general government schemes or compulsory contributory health insurance schemes, (2) domestic health expenditure, which additionally includes private health expenditure (i.e., voluntary health insurance schemes and households' out‐of‐pocket expenditures), and (3) total health expenditure, which additionally includes external aid. Total health expenditure includes all current expenditure related with the provision of health services, family planning activities, nutrition activities, as well as emergency aid designated for health (capital expenditure is not included). While FFS may primarily impact government health expenditure by directly affecting government finances, private health expenditure, and even external aid, may also be affected. For example, households' out‐of‐pocket expenditures may increase to compensate for lower public healthcare provision or may decrease if co‐payments for certain healthcare services become unaffordable. We considered all three health expenditure variables for completeness. All three variables are measured in constant US dollars per capita in the main analyses, or in percent of GDP in some descriptive analyses.

### 3.4 Covariates

The conceptual framework described above informed selection of covariates. *Government finances* were captured through general government expenditures (which must equal government revenues plus borrowing but had fewer missing values) (IMF, n.d.). *Economic activity* was captured by CO_2_ emissions and GDP, and *population health* through mortality from road traffic injury and from non‐communicable diseases (two types of mortality more closely linked with traffic and pollution associated with fossil fuel consumption). All variables and their sources are described in Appendix (Supporting Information S1: Table A1); they were all converted to per capita amounts. Monetary values were measured in constant US dollars.

### 3.5 Descriptive Analyses

We started by characterizing the sample in terms of FFS and health expenditure, per capita or in percent of GDP, across countries over time. We present graphically key bivariate relationships, distinguishing between energy importing (net energy imports > 0) and energy exporting countries (net energy imports < 0). Net energy imports were calculated as the difference between total energy imports and total energy exports, which are both available from the International Energy Agency (IEA)'s Energy Statistics. Importing and exporting countries may have predominantly different support mechanisms underlying their estimated FFS. For example, in exporting countries, forgone corporate taxes and other forms of support for producers are probably more relevant than in importing countries (OECD 2023). We also contrasted countries' spending on FFS and their scores on WHO's UHC Service Coverage Index.

### 3.6 Empirical Model

To determine if FFS crowd out health expenditure, we estimated a panel data model where health expenditure per capita in country i in year t (${HE}_{it}$) is regressed on FFS per capita, controlling for several covariates ($X_{it}$), a time trend (t), and country fixed effects ($C_{i}$) (Equation 1).

(1) $${HE}_{it}=\alpha +\beta FFS_{it}+\gamma X_{it}+t+C_{i}+\varepsilon_{it}$$

We considered government, domestic, and total health expenditure in separate regressions. The country fixed effects capture a variety of country characteristics that may remain relatively constant over a 5‐year period, for example, health system features, infrastructure, political regime, population age structure. In subgroup and sensitivity analyses we further investigated the role of such factors. We tested alternative ways to control for underlying global trends in health expenditure and settled on the parsimonious linear time trend t (see below). Lastly, all variables entered regressions as natural logarithms, to account for skewness of the distributions and reduce the influence of outliers (i.e., log‐log model). As a result, coefficients are interpreted as elasticities (e), that is, they give the percent change in health expenditure per capita associated with a 1% change in the corresponding right‐hand side variable.

### 3.7 Endogeneity and Instrumental Variable Strategy

The included covariates, country fixed effects, and trend may not fully account for all factors potentially affecting health expenditure and correlating with FFS, to allow identification of the causal impact of FFS on health expenditure (i.e., endogeneity bias). The main threat of bias comes from government policy regarding FFS (e.g., deciding to abandon a fixed price mechanism). Such decisions may be correlated with, or driven by, decisions regarding other areas of government spending, including health. For example, governments may be unable to finance themselves, and even require a bailout, which may prompt spending cuts across the board (IMF loans are often conditional on FFS reform; IMF 2013). Changes in government may also simultaneously influence FFS and health expenditure (Droste et al. 2024). Sudden rises in health expenditure, such as those caused by pandemics, could also potentially drive FFS cuts (i.e., reverse causality).

Recalling the way FFS are estimated by the IMF, most components of supply costs are likely to be unrelated with health expenditure or captured in the country fixed effects and trend. Units consumed may change because of changing prices; this will be largely captured by the GDP and CO_2_ emissions variables. However, end‐user prices may be affected by remaining confounding bias from the factors discussed above.

To address this concern, we employed an instrumental variable (IV) strategy. This consists of using an external or exogeneous variable (i.e., IV) that shifts FFS but does not affect health expenditure except through the FFS channel, at least when adjusting for covariates and fixed effects (i.e., effectively creating a quasi‐experimental setting; see e.g., Radulescu and Sulger 2022; Brückner et al. 2012 for related IV applications). To create an IV, we combined two variables to capture a country's vulnerability to international energy prices (Equation 2).

(2) IV=Ln(2010−14averageenergyimports×crudeoilprice)

The premise is that international energy prices can abruptly change, increasing suddenly the cost of keeping domestic energy prices low (i.e., low pass‐through to consumers, Coady et al. 2015). This affects especially countries that import more energy. For example in 2018, when oil prices trended higher than in previous years, FFS rose by 30% (IEA 2019). FFS respond strongly to international energy prices because of instated mechanisms like fixed end‐user prices (e.g., in several Middle Eastern countries), as well as ad hoc (temporary) measures to shield consumers from abrupt energy cost increases, as happened in 2022 across Europe (IEA 2023). In fact, rising oil prices have been identified as a main driver of FFS reform, because of their drastic impact on FFS spending, in both energy importing and exporting countries (IMF 2013). Note that even net energy exporting countries import energy, due to specific energy needs, infrastructure and distribution factors, economic and trade agreements, and other reasons. Both total energy imports and the international crude oil price are strongly and positively correlated with FFS (Supporting Information S1: Figures A4 and A5 in Appendix).

To ensure that total energy imports are unrelated with current health expenditure, we computed the yearly average over the five years preceding the sample period, 2010–2014. So, this variable only varies across countries. Multiplying by the international crude oil price brings in longitudinal variation.

Crucially, the IV is likely to be unrelated with health expenditure except through FFS, conditional on the covariates included in the models and the country fixed effects. For example, the models rule out an effect of international oil prices on health expenditure mediated by changes in economic activity, by controlling for GDP. In fossil fuel‐rich countries, fossil fuel rents are likely related with energy imports and could affect health expenditure through fiscal space. This is mitigated by controlling for both fossil fuel reserves (i.e., country fixed effects) and fiscal space (i.e., general government expenditure), and by using a measure of historic imports (2010–2014 average) rather than current imports.

In summary, to capture the causal impact of increasing FFS on health expenditure, we rely on changes in FFS caused by rising international energy prices, weighed by countries' vulnerability to those prices through energy imports. The main limitation of this IV is the limited longitudinal variation, coming only from variations in the international crude oil price, which are the same for all countries. This precluded the inclusion of year fixed effects in the regressions, which would provide a more flexible specification of global trends. To be able to test the robustness of our results to the inclusion of year fixed effects, we considered two alternative IVs that vary longitudinally within countries.

The first alternative IV is

(3) IV1=Ln(Currentenergyimports×crudeoilprice)

Compared to our preferred IV using pre‐sample values of energy imports (Equation 2), this IV using current energy imports tends to be less strongly associated with FFS, which results in estimated elasticities of health expenditure with respect to FFS that are less precise (Supporting Information S1: Table A8 in Appendix). Nevertheless, results are generally concordant in terms of magnitude. Compared to pre‐sample 2010–14 average energy imports, current energy imports are also potentially less likely to be exogeneous to health expenditure (conditional on all covariates and fixed effects), as is required of an IV.

The second alternative IV is given by:

(4) IV2=Ln(AverageFFSperinhabitantintheregion)

The average is taken across neighboring countries, excluding each country at a time (i.e., leave‐one‐out average), with neighbors being countries in the same WHO region (South‐East Asia, Africa, Americas, Europe, Western Pacific, or Eastern Mediterranean) (Ebeke and Ngouana 2015). There is a strong positive correlation between FFS in a given country and FFS in that country's region (Supporting Information S1: Figure A6 in Appendix). Intuitively, this IV should be exogenous to health expenditure in a given country, as long as shocks affecting multiple neighboring countries are appropriately captured by the trend. Using this alternative IV gives highly concordant findings in terms of both magnitude and statistical significance, across a variety of trend specifications (Supporting Information S1: Table A8 in Appendix).

Durbin‐Wu‐Hausman test results indicated that FFS is endogeneous in almost all regressions, indicating probable bias in models estimated via ordinary least squares (OLS). Therefore, results presented below are from IV regressions (estimated via two‐stage least squares), with OLS results presented in Appendix (Supporting Information S1: Table A6) for comparison. We also show the results of the first stage regressions, which indicate that our preferred IV strongly explains FFS, with F‐statistics well above the common rule‐of‐thumb of 10 (Angrist and Pischke 2008).

### 3.8 Additional Analyses and Robustness Checks

We considered four different sample stratifications. First, we distinguished between energy importing and exporting countries, as their FFS may look very different, not only in terms of volume but also the kinds of fuels subsidized and subsidy mechanisms (see Appendix, Table A2, for the classification). Second and related to FFS volume, we also split the sample into below and above median FFS per capita. Third, we looked separately at LMICs and high‐income countries (HICs), based on the World Bank's classification. Fourth, to investigate the role of health system type, we split the sample into countries with predominantly government‐, social health insurance‐, or out‐of‐pocket‐funded health systems, based on the classification developed by Gabani et al. (2023).

We also conducted several sensitivity analyses. We replicated the main models using only subsidies to petroleum products instead of total FFS, which may be more responsive to international crude oil prices (our IV) than coal, natural gas, and electricity subsidies. We excluded (some) covariates, replaced government expenditures per capita with the sum of government revenues and net borrowing (which are two sides of the government budget equation), and included additional covariates capturing the proportions of the population under 15 years of age and 65 and above, whether the country is democratic and the left or right wing inclination of the leading party (Herre 2023). These additional covariates were not included in the main models to reduce multicollinearity and maximize sample size. We also fitted alternative specifications of the trend (region‐specific linear trends, quadratic trend, year fixed effects), used alternative IVs (see previous section), and attenuated outliers by setting values below the fifth(/first) and above the 95^th^(/99^th^) percentiles to the fifth(/first) and 95^th^(/99^th^) percentile values (i.e., winsorization).

## 4 Results

The study analyzed 82 energy‐importing and 44 energy‐exporting countries, as detailed in Appendix (Supporting Information S1: Table A2). On average, countries spent $210 per capita on FFS annually during 2015–2019, while total health expenditure per capita averaged $1437. Both variables exhibited highly right‐skewed distributions, especially FFS per capita, with significant variation across countries. The standard deviation and maximum values of FFS per capita were $476 and $5943 ($2102 and $10,790 for total health expenditure per capita). Additional summary statistics are provided in Table A1 in Appendix.

**FIGURE 2:**
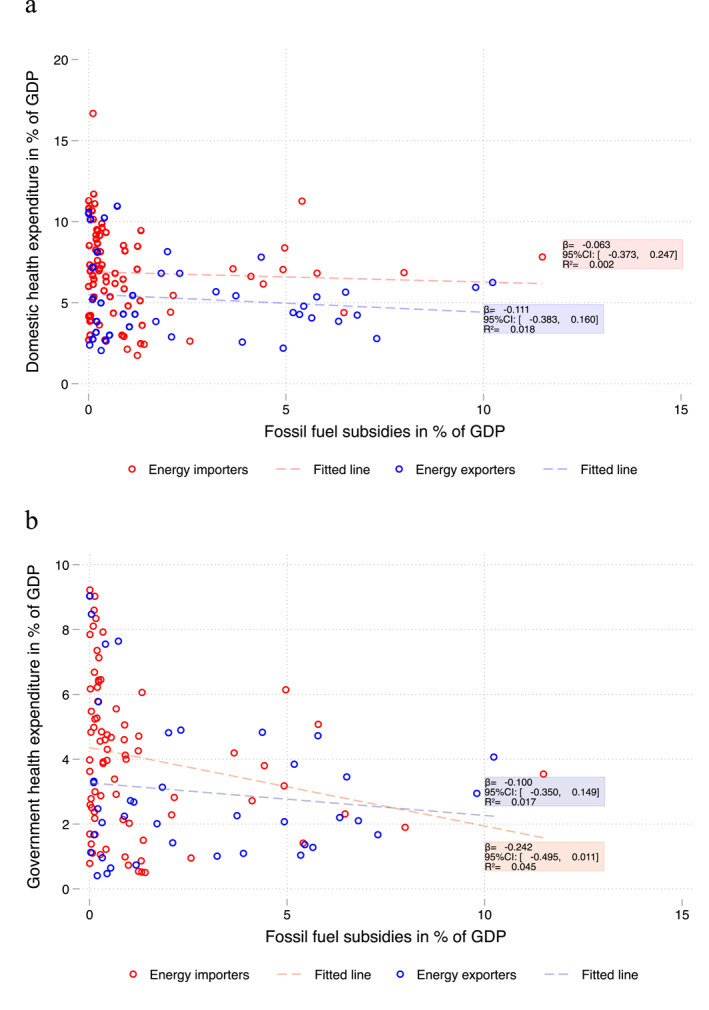
(a) Domestic health expenditure and FFS in % of GDP, 2019. (b) Government health expenditure and FFS in % of GDP, 2019. Excludes Venezuela (outlier with FFS > 30% GDP). *Source:* own elaboration based on IMF FFS estimates and WHO's Global Health Expenditure Database.

**FIGURE 3:**
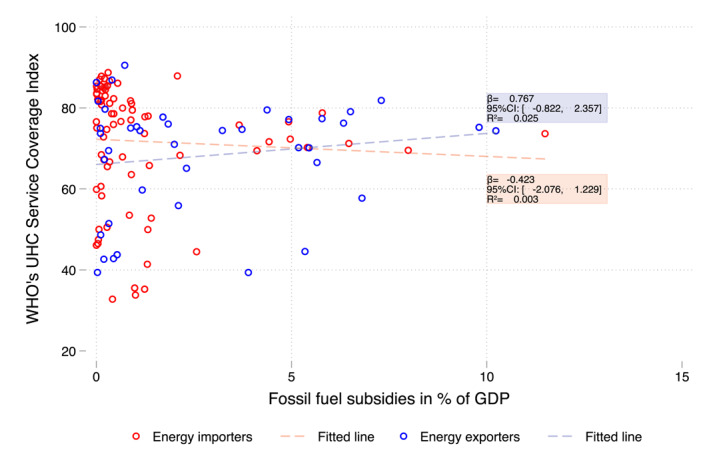
WHO's UHC Service Coverage Index and FFS in % of GDP, 2019. Excludes Venezuela (outlier with FFS > 30% GDP). *Source:* Own elaboration based on IMF FFS estimates and WHO's UHC Service Coverage Index.

A small negative correlation was observed between FFS and health expenditure as percentages of GDP, based on cross‐country variations in 2019 (Figure 2). Seventeen countries (Algeria, Armenia, Azerbaijan, Bahrain, Egypt, Estonia, Iran, Iraq, Kazakhstan, Kuwait, Kyrgyzstan, Lebanon, Oman, Russia, Sudan, Tajikistan, and Venezuela) spent more than 5% of their GDP on FFS, most of which were net energy exporters. Notably, 15 of these 17 countries (all except Armenia and Estonia) spent a higher percentage of GDP on FFS than on health (considering domestic health expenditure, Figure 2a). Moreover, all but Kazakhstan among these 17 countries scored below 80 on the WHO's UHC service coverage index in 2019 (Figure 3), although no clear linear correlation was found between UHC scores and FFS as a percentage of GDP across countries.

Between 2015 and 2019, there were no consistent upward or downward trends in average FFS or health expenditure per capita among either energy‐importing or exporting countries (Supporting Information S1: Appendix Figures A1‐A3). The trend in domestic health expenditure closely followed that of FFS in both country groups (Supporting Information S1: Appendix Figure A1). Energy‐exporting countries generally exhibited higher FFS levels (statistically significant in most years at the 95% confidence level) and lower health expenditure per capita (with overlapping 95% confidence intervals), compared to importing countries. This reinforces the need to separately analyze these two groups of countries and include country fixed effects in the regressions.

Our IV results for all countries showed that, after adjusting for covariates, fixed effects and trend, a 1% increase in FFS per capita (triggered by rising international oil prices) led to a 0.052% decrease in domestic health expenditure per capita (95% CI −0.084% to −0.020%; Table 1). At the sample averages, this implies that a $1 increase in FFS per capita corresponds to a $0.35 decrease in domestic health expenditure per capita (−0.052%*$1422.37/1%*$210.16 = $−0.35). The elasticity was similar for total health expenditure (−0.045%; 95% CI −0.076% to −0.014%) and slightly smaller for government health expenditure (−0.038%; 95% CI −0.080%−0.003%).

**TABLE 1:** Impacts of FFS on total, domestic, and government health expenditure (IV results).

		Total HE	Domestic HE	Government HE
	Ln(FFS pc)	Ln(HE pc)	Ln(HE pc)	Ln(HE pc)
Ln(FFS pc)		−0.045***	−0.052***	−0.038*
		[−0.076,−0.014]	[−0.084,−0.020]	[−0.080,0.003]
Ln(general government expenditure pc)	−0.952*	0.081	0.057	0.275
	[−2.016,0.112]	[−0.108,0.269]	[−0.137,0.250]	[−0.055,0.605]
Ln(CO_2_ emissions pc)	0.817	0.020	0.013	−0.017
	[−0.263,1.898]	[−0.133,0.173]	[−0.145,0.170]	[−0.236,0.202]
Ln(GDP pc)	0.850*	0.849***	0.871***	0.692***
	[−0.137,1.837]	[0.659,1.039]	[0.673,1.069]	[0.356,1.028]
Ln(Mortality from road traffic injury)	0.459	0.036	0.040	0.137
	[−0.281,1.200]	[−0.083,0.155]	[−0.078,0.157]	[−0.050,0.323]
Ln(Mortality from NCD)	−1.415	0.066	0.001	0.281
	[‐4.615,1.784]	[−0.547,0.679]	[−0.605,0.606]	[−0.484,1.047]
Ln(Total energy imports*oil price)	1.360***			
	[0.913,1.807]			
Trend	0.029	0.007	0.007	0.014
	[−0.050,0.108]	[−0.006,0.019]	[−0.006,0.020]	[−0.006,0.034]
Country FE	Yes	Yes	Yes	Yes
Instrument strength test (first stage F‐statistic)	F(1,119) = 36.29***			
Durbin‐Wu‐Hausman endogeneity test		Chi‐sq(1) = 8.888***	Chi‐sq(1) = 12.031***	Chi‐sq(1) = 2.163
# Observations	567	567	567	567
# Countries	120	120	120	120

*Note:* 95% confidence intervals in brackets based on standard errors clustered at the country level.

Abbreviations: FE = fixed effects. FFS = fossil fuel subsidies. GDP = gross domestic product. HE = health expenditure. IV = instrumental variable. NCD = non‐communicable diseases. pc = per capita.

^*^ *p* < 0.1.

**p < 0.05.

^***^ *p* < 0.01.

Table 2 presents the regression results for different country groups. The negative relationship between FFS and domestic health expenditure per capita was consistent across energy importing and exporting countries, appeared stronger in HIC (e∼0.06%, 95% CI −0.140%–0.012%) than in LMIC (e∼0.04%, 95% CI −0.080% to −0.005%), and was more pronounced in countries with above‐median FFS per capita (e∼0.07% vs. e∼0.03%). The main effect was driven by countries with health systems funded predominantly by out‐of‐pocket payments and government financing; in social health insurance‐funded systems, the elasticity was near zero. However, these stratified analyses lacked sufficient power to detect statistically significant differences between the groups. Similar results were observed for total and government health expenditure (Supporting Information S1: Appendix Tables A3 and A4).

**TABLE 2:** Impacts of FFS on domestic health expenditure, energy importing v. exporting countries, LMICs v. HICs, low v. high FFS, government‐v. social health insurance‐v. out‐of‐pocket‐funded health systems (IV results).

	Energy importers	Energy exporters	LMIC	HIC	Low FFS	High FFS	GOV	SHI	OOP
	Ln(HE pc)	Ln(HE pc)	Ln(HE pc)	Ln(HE pc)	Ln(HE pc)	Ln(HE pc)	Ln(HE pc)	Ln(HE pc)	Ln(HE pc)
Ln(FFS pc)	−0.052**	−0.054**	−0.042**	−0.064*	−0.029*	−0.068**	−0.058***	−0.003	−0.060*
	[−0.103,−0.001]	[−0.097,−0.010]	[−0.080,−0.005]	[−0.140,0.012]	[−0.063,0.005]	[−0.132,−0.004]	[−0.100,−0.016]	[−0.046,0.039]	[−0.130,0.011]
Ln(general government expenditure pc)	0.081	−0.000	0.037	0.240	−0.133	0.350***	0.232	0.347**	−0.026
	[−0.237,0.400]	[−0.265,0.265]	[−0.166,0.240]	[−0.137,0.617]	[−0.326,0.060]	[0.151,0.549]	[−0.100,0.563]	[0.078,0.616]	[−0.246,0.194]
Ln(CO2 emissions pc)	−0.084	0.180	0.023	0.124	−0.102	0.205	−0.167	0.050	0.126
	[−0.249,0.081]	[−0.135,0.494]	[−0.169,0.216]	[−0.125,0.373]	[−0.236,0.032]	[−0.099,0.508]	[−0.454,0.121]	[−0.107,0.206]	[−0.160,0.411]
Ln(GDP pc)	0.720***	0.937***	0.891***	0.831*	1.324***	0.588***	0.568	0.295	0.962***
	[0.224,1.217]	[0.666,1.208]	[0.681,1.101]	[−0.040,1.702]	[0.742,1.905]	[0.398,0.778]	[−0.185,1.320]	[−0.205,0.794]	[0.740,1.183]
Ln(Mortality from road traffic injury)	0.037	0.032	0.093	−0.062	−0.041	0.033	−0.020	0.028	0.106
	[−0.063,0.138]	[−0.280,0.343]	[−0.034,0.220]	[−0.204,0.081]	[−0.144,0.062]	[−0.165,0.230]	[−0.274,0.234]	[−0.060,0.116]	[−0.101,0.312]
Ln(Mortality from NCD)	0.204	−0.376	0.258	−0.391	−0.445*	0.104	−0.142	−0.326	−0.105
	[−0.450,0.857]	[−1.288,0.535]	[−0.396,0.912]	[−1.344,0.562]	[−0.956,0.065]	[−0.687,0.896]	[−1.353,1.070]	[−0.991,0.339]	[−1.173,0.963]
Trend	0.016**	−0.003	0.006	0.004	−0.004	0.010	0.004	0.012	−0.006
	[0.002,0.030]	[−0.027,0.022]	[−0.008,0.020]	[−0.017,0.025]	[−0.021,0.013]	[−0.005,0.026]	[−0.020,0.027]	[−0.009,0.033]	[−0.027,0.016]
Country FE	Yes	Yes	Yes	Yes	Yes	Yes	Yes	Yes	Yes
Instrument strength test (first stage F‐statistic)	F(1,77) = 13.61***	F(1,41) = 22.92***	F(1,75) = 25.09***	F(1,43) = 9.35***	F(1,61) = 14.56***	F(1,57) = 37.63***	F(1,32) = 13.48***	F(1,30) = 7.76***	F(1,49) = 9.51***
Durbin‐Wu‐Hausman endogeneity test	Chi‐sq(1) = 6.003**	Chi‐sq(1) = 6.259**	Chi‐sq(1) = 4.729**	Chi‐sq(1) = 5.002**	Chi‐sq(1) = 2.792*	Chi‐sq(1) = 5.436**	Chi‐sq(1) = 6.598**	Chi‐sq(1) = 0.341	Chi‐sq(1) = 4.856**
# Observations	368	199	351	216	283	284	161	155	222
# Countries	78	42	76	44	62	58	33	31	50

*Note:* 95% confidence intervals in brackets based on standard errors clustered at the country level.

Abbreviations: FE = fixed effects. FFS = fossil fuel subsidies. GDP = gross domestic product. GOV = predominantly government‐funded health system. HE = health expenditure. HICs = high income countries. IV = instrumental variable. LMICs = low‐ and middle‐income countries. NCD = non‐communicable diseases. OOP = predominantly out‐of‐pocket‐funded health system. pc = per capita. SHI = predominantly social health insurance‐funded health system. Classification into predominantly government‐, social health insurance‐, or out‐of‐pocket‐funded health systems available in Gabani et al. (2023).

^*^ *p* < 0.1.

^**^ *p* < 0.05.

^***^ *p* < 0.01.

Finally, our results remained robust when restricting the analysis to subsidies for petroleum products, excluding covariates like general government expenditure per capita and mortality from non‐communicable diseases, or including the additional covariates described above (Supporting Information S1: Appendix Tables A5‐A7). Alternative trend specifications and IVs yielded consistent findings (Supporting Information S1: Appendix Table A8). The results also held when applying winsorization at the top and bottom percentiles (1% and 5%; Supporting Information S1: Appendix Table A9).

## 5 Discussion

This study found that in many countries, FFS spending exceeds health expenditures, with potential negative implications for achieving UHC. Specifically, a 1% increase in FFS per capita results in a 0.052% reduction in domestic health expenditure per capita. At the mean, this translates to a crowding out effect where each additional dollar spent on FFS reduces domestic health expenditures per capita by $0.35, or $0.18 when considering only government health expenditure. These findings align with and expand upon previous studies that focused on a smaller subset of countries (Klaiber et al. 2023; Romanello et al. 2022; Gupta et al. 2015; Ebeke and Ngouana 2015), suggesting that eliminating FFS could be a vehicle to advance UHC as well as other Sustainable Development Goals (UNDP 2019).

To put our estimated elasticity of −0.05% into perspective, consider that the mean, median, and 75^th^ percentile annual growth rates in FFS per capita during 2015‐19 were 163%, 4%, and 44%, respectively. These figures contrast with much lower annual growth rates in domestic health expenditure per capita of 13%, 2%, and 5% over the same period. The estimated elasticities for total, domestic, and government health expenditure were not statistically different, though the elasticity for government health expenditure was almost as large in absolute terms, suggesting that government expenditure is a key driver of these results. We also found that the effects were more pronounced in countries with predominantly government‐funded health systems, despite the fact that governments often have other, potentially more flexible, options for financial adjustment, such as borrowing, issuing money, raising taxes, delaying large infrastructure projects, or cutting spending in other sectors (note that our models control for government finances through general government expenditures; Ortiz, Cummins, and Karunanethy 2017). Notably, the elasticities did not differ significantly between energy importing and exporting countries, or between HIC and LMIC.

This study makes two major contributions. First, by compiling data from the IMF and WHO for most of the world's countries, we provide a comprehensive global analysis of the relationship between FFS and health expenditure. Second, by employing a novel IV approach to capture sudden (exogeneous) changes in FFS, we present causal evidence that rising FFS crowds‐out health expenditure. Our IV approach also empirically confirms that both net energy importing and exporting countries that subsidize fossil fuels are vulnerable to international energy prices (Al‐Saidi 2020). Additionally, our findings underscore the utility of IMF FFS estimates for empirical work, particularly their link with government finances, and show that a combination of countries' energy imports and international oil prices can serve as a valid IV for FFS in future studies.

However, our study is not without limitations. IMF estimates of FFS rely on various assumptions, such as transportation costs and distribution margins, and are based on end‐user prices at the end of the year (IMF 2013). Nevertheless, they remain the most comparable estimates across a larger number of countries. The alternative approach to measure FFS (OECD's inventory) covers only 51, mostly developed, economies (the inventory documents specific government support measures for fossil fuel production or consumption, such as transfers of government funds to compensate producers for price controls, forgone fiscal revenue from waived taxes, subsidized inputs, and underpricing of permits, some of which are not easily quantifiable; Kojima and Koplow 2015). Another limitation is that IMF FFS data have only been available since 2015, limiting the time horizon for the analysis. Crucially, this precluded the exploration of dynamic and long‐term effects. Underlying dynamic effects may introduce confounding, but this threat is likely to be limited, because we rely on changes in FFS expenditure that result from international oil price shocks to capture short‐term adjustments in health expenditure. Expanding the time horizon to more recent years is not without challenges due to the potentially confounding effects of the COVID‐19 pandemic. Importantly, the subsidies considered in this study reflect only the mechanisms in place to keep consumer prices below supply costs (i.e., explicit subsidies). These represent only a small fraction of the true total cost of subsidizing fossil fuels, which also include underpricing of air pollution, climate change, and other externalities (i.e., implicit subsidies). When those are accounted for, the total estimate of FFS rises to $7 trillion in 2022 (Black et al. 2023). A drawback of our IV approach is that longitudinal variation comes only from annual changes in the crude oil price, which are uniform across countries. This precluded the inclusion of year fixed effects in our models due to multicollinearity with the IV and resulting loss of statistical power. However, our results were robust to alternative IVs that varied over time and allowed for the inclusion of year fixed effects.

Overall, our findings showed that across different contexts and over time, high levels of FFS are often associated with relatively low health expenditure and limited health service coverage, as measured by the WHO's UHC Service Coverage Index. We also showed that FFS expansions have crowded out health expenditure at an average elasticity of −0.05% during 2015–2019. This suggests that cutting FFS could free up resources for expansions in UHC. Our estimate is a net elasticity, suggesting that any potentially favorable indirect effects of FFS, through protecting economic activity or population health, are negligible compared to the direct effects on government finances. In fact, our result that both public and private health expenditure are negatively affected by increases in FFS suggests that there are no favorable effects of FFS. This finding is consistent with households decreasing healthcare utilisation because healthcare services are less widely available or co‐payments become unaffordable. However, these results pertain to aggregate expenditure and may mask unequal impacts across different socioeconomic groups, an area that deserves further investigation. Additionally, a comprehensive investigation of the welfare implications of FFS should include other dimensions of social spending, including education and social protection—are all areas similarly affected, or is health more or less prone to budget cuts following FFS increases? We refrained from considering other expenditure outcomes ad hoc, without a well‐developed conceptual framework. A descriptive exercise based on the government budget equation (i.e., fiscal identity) could provide additional insights (i.e., looking at changes in each source of government revenue, each type of expenditure, and borrowing/lending). However, this exercise may only be possible for single countries, given limitations with national accounts data and the fact that FFS data are based on price‐gap estimations that don't directly map onto governments' accounts.

Over 50 countries undertook some form of FFS reform since 2015 and there was a renewed commitment to eliminate FFS at the Summit of the Future in September 2024 (Sanchez et al. 2020; United Nations 2024). Simply cutting FFS could harm the poor and increase inequalities, however reinvesting FFS savings into appropriate pro‐poor social spending, including health sector investments, is likely to have overall benefits (Webb et al. 2023; Romanello et al. 2022; Alers and Jones 2021; Gupta et al. 2015; del Granado et al. 2012; Rentschler 2016; Rentschler and Bazilian 2017; Mokhtari and Ghoddusi 2025). Appropriate and well communicated social investments may also enhance the feasibility of reform by reducing public opposition (McCulloch et al. 2022; del Granado et al. 2012; Uexkull et al. 2024). Future research should explore the impacts of recent reforms, as well as earlier and more ambitious ones, such as Indonesia's successive reforms since 1997 (Chelminski 2018), on countries' progress toward UHC and on population health outcomes. The political economy of cutting FFS and investing in health and other social spending is also worthy of further investigation.

In summary, FFS exceeded $1 trillion in 2022, representing a 100% increase from the previous year (Black et al. 2023). Our findings indicate that beyond the role in limiting global temperature increases to 1.5°C, eliminating FFS could free up substantial domestic resources. These are much needed to achieve UHC and SDG3, especially in an era of steep decline in international aid for health (Apeagyei et al. 2025).

## Author Contributions

**Judite Gonçalves:** conceptualization, methodology, data curation, analysis, paper drafting, reviewing, and editing. **Eduardo Costa:** conceptualization, methodology, reviewing, and editing. **Thomas Hone:** conceptualization, methodology, reviewing, and editing. **Damini Singh:** methodology, reviewing, and editing. **Paula Pereda:** methodology, reviewing, and editing. **Anthony A. Laverty:** methodology, reviewing, and editing. **Christopher Millett:** conceptualization, data curation, paper drafting, reviewing, and editing.

## Conflicts of Interest

The authors declare no conflicts of interest.

## Supporting information


Supporting Information S1

## Data Availability

The data that support the findings of this study are available from public sources detailed in Table A1 in the Supplementary Materials.
