# Supplementary material for: Do Fossil Fuel Subsidies Crowd Out Health Expenditure? A Country‐Level Longitudinal Analysis
Source: Health Econ. 2025 Dec 20;35(4):677–87. doi: 10.1002/hec.70074 (PMC12950206; doi:10.1002/hec.70074)
Supplement: Supplementary file 1 — Supporting Information S1 [file HEC-35-677-s001.docx]

**Supplementary Material**

**Figures**

|  |  |
| --- | --- |
| Figure A1. Evolution of FFS and domestic health expenditure, 2015-2019 | |
| Notes: Mean of FFS per inhabitant and domestic health expenditure per inhabitant over all energy importing or exporting countries.  Source: own elaboration based on IMF FFS estimates and WHO’s Global Health Expenditure Database. | |

Figure A2. Evolution of FFS in energy importing v. energy exporting countries, 2015-2019

Notes: Mean of FFS per inhabitant over all energy importing or exporting countries.

Source: own elaboration based on IMF FFS estimates.

Figure A3. Evolution of domestic health expenditure in energy importing v. energy exporting countries, 2015-2019

Notes: Mean of domestic health expenditure per inhabitant over all energy importing or exporting countries.

Source: own elaboration based on WHO’s Global Health Expenditure Database.

|  |  |
| --- | --- |
| Figure A4. Evolution of FFS and crude oil prices, 2015-2019 | |
| Notes: Mean of FFS per inhabitant over all energy importing or exporting countries.  Source: own elaboration based on IMF FFS estimates and crude oil price data from bp’s Statistical Review of World Energy June 2022. | |

|  |
| --- |
| Figure A5. FFS in 2019 and total energy imports per inhabitant (2010-14 average) |
| Source: own elaboration based on IMF FFS estimates and IEA energy statistics. |

Figure A6. FFS in the country and FFS in the region in 2019

Notes: Average FFS per inhabitant across neighbouring countries, excluding each country a time (i.e., leave-one-out average). From left to right (i.e., increasing average FFS), the WHO region clusters are: South-East Asia, Africa, Americas, Europe, Western Pacific, Eastern Mediterranean.

Source: own elaboration based on IMF FFS estimates.

**Tables**

Table A1. Variables, descriptive statistics, and sources

| **Variable & Definition** | **Mean** | **Std. Dev.** | **Min.** | **Max.** | **Data Source** |
| --- | --- | --- | --- | --- | --- |
| FFS, 2021 USD per inhabitant (Explicit subsidies, all fuels) | 210.16 | 476.43 | 0.01 | 5,943.32 | IMF FFS by Country and Fuel Database, https://www.imf.org/en/Topics/climate-change/energy-subsidies |
| Total health expenditure, 2020 USD per inhabitant | 1,437.39 | 2,102.40 | 7.92 | 10,789.52 | WHO’s Global Health Expenditure Database, apps.who.int/nha/database |
| Domestic health expenditure, 2020 USD per inhabitant | 1,433.37 | 2,101.72 | 7.92 | 10,789.52 |  |
| Government health expenditure, 2020 USD per inhabitant | 969.50 | 1,456.28 | 2.18 | 6,183.52 |  |
| General government expenditure, 2020 USD per inhabitant | 6,870.98 | 9,211.05 | 62.39 | 52,151.08 |  |
| GDP, 2020 USD per inhabitant | 17,824.61 | 21,756.60 | 182.17 | 121,648.80 |  |
| CO_2_ emissions, metric tons per inhabitant | 5.59 | 5.52 | 0.08 | 35.11 | World Bank’s World Development Indicators, databank.worldbank.org/source/world-development-indicators |
| Mortality from road traffic injury per 100,000 inhabitants | 14.45 | 8.52 | 2.10 | 64.60 |  |
| Mortality from non-communicable diseases, age-standardised, per 100,000 inhabitants | 517.98 | 158.32 | 233.30 | 960.80 | World Health Organization’s Global Health Observatory, https://www.who.int/data/gho/data/indicators/indicator-details/GHO/gho-ghe-ncd-mortality-rate |
| Total energy imports, TJ per inhabitant (2010-2014 yearly average) | 0.07 | 0.14 | 0.00 | 1.24 | IEA Energy Statistics Data Browser, https://www.iea.org/data-and-statistics/data-tools/energy-statistics-data-browser |
| Annual spot crude oil price, 2020 USD per barrel | 57.60 | 8.38 | 45.04 | 70.20 | Bp Statistical Review of World Energy June 2022, http://www.bp.com/statisticalreview |

Table A2. List of included countries

| **Energy importers (n=82)** | Estonia | Lithuania | Slovakia | Bahrain | Myanmar |
| --- | --- | --- | --- | --- | --- |
| Argentina | Ethiopia | Luxembourg | Slovenia | Bolivia | Nigeria |
| Armenia | Finland | Madagascar | South Korea | Brunei | Norway |
| Austria | France | Mauritius | Spain | Cameroon | Oman |
| Bangladesh | Georgia | Moldova | Sri Lanka | Canada | Paraguay |
| Belarus | Germany | Morocco | Suriname | Colombia | Peru |
| Belgium | Ghana | Nepal | Sweden | Congo | Qatar |
| Benin | Greece | Netherlands | Switzerland | Denmark | Russia |
| Bosnia and Herzegovina | Guatemala | New Zealand | Tajikistan | Ecuador | Saudi Arabia |
| Brazil | Haiti | Nicaragua | Thailand | Egypt | South Africa |
| Bulgaria | Honduras | Niger | Togo | Equatorial Guinea | Sudan |
| Cambodia | Hungary | North Macedonia | Tunisia | Gabon | Trinidad and Tobago |
| Chile | India | Pakistan | Turkey | Indonesia | Turkmenistan |
| China | Ireland | Panama | Ukraine | Iran | United Arab Emirates |
| Costa Rica | Israel | Philippines | United Kingdom | Iraq | Uzbekistan |
| Croatia | Italy | Poland | United States of America | Kazakhstan | Venezuela |
| Cyprus | Jamaica | Portugal | Zambia | Kuwait | Vietnam |
| Czech Republic | Japan | Romania | **Energy exporters (n=44)** | Laos | Yemen |
| Cote d'Ivoire | Jordan | Rwanda | Algeria | Malaysia |  |
| Dominican Republic | Kyrgyzstan | Senegal | Angola | Mexico |  |
| El Salvador | Latvia | Serbia | Australia | Mongolia |  |
| Eritrea | Lebanon | Singapore | Azerbaijan | Mozambique | |

Notes: Countries are excluded due to missing information on any of the included variables (mostly small and island countries, which are usually excluded in analyses of health expenditure on account of their peculiarities with respect to health system features and financing),(Gabani, Mazumdar, and Suhrcke 2023) or due to estimated FFS=0 (ln not defined). Countries highlighted in grey are those that subsidize petroleum products, analysed in a sensitivity check.

Table A3. Impacts of FFS on total health expenditure, energy importing v. exporting countries, LMICs v. HICs, low v. high FFS, government- v. social health insurance- v. out-of-pocket-funded health systems (IV results)

|  | **Energy importers** | **Energy exporters** | **LMIC** | **HIC** | **Low FFS** | **High FFS** | **GOV** | **SHI** | **OOP** |
| --- | --- | --- | --- | --- | --- | --- | --- | --- | --- |
|  | Ln(HE pc) | Ln(HE pc) | Ln(HE pc) | Ln(HE pc) | Ln(HE pc) | Ln(HE pc) | Ln(HE pc) | Ln(HE pc) | Ln(HE pc) |
| Ln(FFS pc) | -0.029 | -0.054** | -0.033* | -0.063 | -0.019 | -0.066** | -0.056*** | -0.003 | -0.047 |
|  | [-0.077,0.019] | [-0.098,-0.010] | [-0.069,0.004] | [-0.138,0.013] | [-0.053,0.015] | [-0.130,-0.003] | [-0.096,-0.017] | [-0.046,0.041] | [-0.115,0.021] |
| Ln(general government expenditure pc) | 0.100 | 0.035 | 0.059 | 0.250 | -0.092 | 0.349*** | 0.139 | 0.356** | 0.015 |
|  | [-0.184,0.383] | [-0.231,0.301] | [-0.138,0.255] | [-0.121,0.622] | [-0.281,0.097] | [0.151,0.547] | [-0.141,0.418] | [0.079,0.634] | [-0.202,0.231] |
| Ln(CO2 emissions pc) | -0.048 | 0.166 | 0.040 | 0.123 | -0.067 | 0.176 | -0.061 | 0.038 | 0.124 |
|  | [-0.214,0.117] | [-0.141,0.473] | [-0.145,0.225] | [-0.124,0.369] | [-0.220,0.087] | [-0.130,0.482] | [-0.344,0.222] | [-0.121,0.198] | [-0.151,0.399] |
| Ln(GDP pc) | 0.619*** | 0.903*** | 0.872*** | 0.815* | 1.268*** | 0.587*** | 0.683** | 0.259 | 0.925*** |
|  | [0.198,1.040] | [0.634,1.172] | [0.672,1.071] | [-0.048,1.677] | [0.743,1.794] | [0.399,0.775] | [0.052,1.313] | [-0.270,0.789] | [0.711,1.139] |
| Ln(Mortality from road traffic injury) | 0.040 | 0.035 | 0.086 | -0.060 | -0.066 | 0.040 | 0.005 | 0.032 | 0.081 |
|  | [-0.057,0.136] | [-0.274,0.344] | [-0.049,0.221] | [-0.202,0.081] | [-0.170,0.038] | [-0.161,0.241] | [-0.248,0.259] | [-0.056,0.119] | [-0.136,0.297] |
| Ln(Mortality from NCD) | 0.260 | -0.331 | 0.371 | -0.385 | -0.403* | 0.152 | -0.178 | -0.286 | 0.047 |
|  | [-0.368,0.888] | [-1.247,0.586] | [-0.301,1.042] | [-1.334,0.564] | [-0.861,0.056] | [-0.655,0.958] | [-1.385,1.029] | [-0.972,0.400] | [-1.044,1.139] |
| Trend | 0.015** | -0.001 | 0.005 | 0.004 | -0.005 | 0.010 | 0.005 | 0.013 | -0.007 |
|  | [0.003,0.028] | [-0.025,0.022] | [-0.008,0.019] | [-0.017,0.025] | [-0.021,0.010] | [-0.005,0.026] | [-0.019,0.029] | [-0.009,0.035] | [-0.027,0.013] |
| Country FE | Yes | Yes | Yes | Yes | Yes | Yes | Yes | Yes | Yes |
| Instrument strength test (first stage F-statistic) | F(1,77)=13.61*** | F(1,41)=22.92*** | F(1,75)=25.09*** | F(1,43)=9.35*** | F(1,61)=14.56*** | F(1,57)=37.63*** | F(1,32)=13.48*** | F(1,30)=7.76*** | F(1,49)=9.51*** |
| Durbin-Wu-Hausman endogeneity test | Chi-sq(1)=1.778 | Chi-sq(1)=6.732*** | Chi-sq(1)=2.951* | Chi-sq(1)=4.805** | Chi-sq(1)=0.801 | Chi-sq(1)=5.283** | Chi-sq(1)=5.776** | Chi-sq(1)=0.332 | Chi-sq(1)=3.271* |
| # Observations | 368 | 199 | 351 | 216 | 283 | 284 | 161 | 155 | 222 |
| # Countries | 78 | 42 | 76 | 44 | 62 | 58 | 33 | 31 | 50 |

Notes: 95% confidence intervals in brackets based on standard errors clustered at the country level. *p<0.1, **p<0.05, ***p<0.01. LMICs=low- and middle-income countries. HICs=high income countries. GOV=predominantly government-funded health system. SHI=predominantly social health insurance-funded health system. OOP=predominantly out-of-pocket-funded health system. HE=health expenditure. pc=per capita. FFS=fossil fuel subsidies. GDP=gross domestic product. NCD=non-communicable diseases. FE=fixed effects. IV=instrumental variable. Classification into predominantly government-, social health insurance-, or out-of-pocket-funded health systems available in Gabani et al. (2023)

Table A4. Impacts of FFS on government health expenditure, energy importing v. exporting countries, LMICs v. HICs, low v. high FFS, government- v. social health insurance- v. out-of-pocket-funded health systems (IV results)

|  | **Energy importers** | **Energy exporters** | **LMIC** | **HIC** | **Low FFS** | **High FFS** | **GOV** | **SHI** | **OOP** |
| --- | --- | --- | --- | --- | --- | --- | --- | --- | --- |
|  | Ln(HE pc) | Ln(HE pc) | Ln(HE pc) | Ln(HE pc) | Ln(HE pc) | Ln(HE pc) | Ln(HE pc) | Ln(HE pc) | Ln(HE pc) |
| Ln(FFS pc) | -0.060 | -0.033 | -0.024 | -0.058 | -0.031 | -0.052 | -0.070** | 0.015 | -0.046 |
|  | [-0.135,0.014] | [-0.088,0.023] | [-0.079,0.031] | [-0.135,0.018] | [-0.083,0.021] | [-0.116,0.013] | [-0.129,-0.011] | [-0.038,0.069] | [-0.133,0.042] |
| Ln(general government expenditure pc) | 0.410*** | 0.156 | 0.248 | 0.444** | 0.082 | 0.524* | 0.274 | 0.366** | 0.288 |
|  | [0.121,0.698] | [-0.324,0.637] | [-0.107,0.604] | [0.030,0.859] | [-0.262,0.425] | [-0.004,1.053] | [-0.215,0.762] | [0.078,0.654] | [-0.083,0.659] |
| Ln(CO2 emissions pc) | -0.164* | 0.161 | -0.026 | 0.204* | -0.277* | 0.366 | -0.098 | 0.006 | -0.023 |
|  | [-0.351,0.024] | [-0.394,0.716] | [-0.307,0.255] | [-0.038,0.445] | [-0.562,0.008] | [-0.170,0.903] | [-0.401,0.204] | [-0.127,0.138] | [-0.420,0.375] |
| Ln(GDP pc) | 0.636** | 0.809*** | 0.718*** | 0.687 | 1.290*** | 0.450* | 0.779 | 0.486** | 0.682*** |
|  | [0.135,1.138] | [0.325,1.294] | [0.352,1.085] | [-0.292,1.665] | [0.343,2.238] | [-0.047,0.947] | [-0.301,1.859] | [0.099,0.873] | [0.310,1.054] |
| Ln(Mortality from road traffic injury) | 0.011 | 0.326 | 0.146 | 0.059 | 0.039 | 0.201 | 0.161* | 0.043 | 0.029 |
|  | [-0.090,0.112] | [-0.161,0.812] | [-0.111,0.403] | [-0.053,0.171] | [-0.099,0.177] | [-0.175,0.577] | [-0.031,0.353] | [-0.059,0.145] | [-0.209,0.267] |
| Ln(Mortality from NCD) | 0.511 | -0.185 | 0.740* | -0.408 | 0.272 | 0.317 | -0.378 | -0.701* | 0.398 |
|  | [-0.224,1.246] | [-1.250,0.880] | [-0.051,1.531] | [-1.522,0.706] | [-0.487,1.031] | [-0.767,1.401] | [-1.766,1.010] | [-1.431,0.029] | [-0.576,1.372] |
| Trend | 0.022** | 0.001 | 0.016 | 0.008 | 0.007 | 0.023 | 0.001 | -0.000 | 0.004 |
|  | [0.002,0.042] | [-0.042,0.044] | [-0.008,0.040] | [-0.017,0.034] | [-0.027,0.040] | [-0.007,0.052] | [-0.031,0.034] | [-0.021,0.021] | [-0.034,0.042] |
| Country FE | Yes | Yes | Yes | Yes | Yes | Yes | Yes | Yes | Yes |
| Instrument strength test (first stage F-statistic) | F(1,77)=13.61*** | F(1,41)=22.92*** | F(1,75)=25.09*** | F(1,43)=9.35*** | F(1,61)=14.56*** | F(1,57)=37.63*** | F(1,32)=13.48*** | F(1,30)=7.76*** | F(1,49)=9.51*** |
| Durbin-Wu-Hausman endogeneity test | Chi-sq(1)=2.674 | Chi-sq(1)=0.547 | Chi-sq(1)=0.242 | Chi-sq(1)=3.720* | Chi-sq(1)=0.586 | Chi-sq(1)=3.139* | Chi-sq(1)=5.019** | Chi-sq(1)=0.503 | Chi-sq(1)=1.796 |
| # Observations | 368 | 199 | 351 | 216 | 283 | 284 | 161 | 155 | 222 |
| # Countries | 78 | 42 | 76 | 44 | 62 | 58 | 33 | 31 | 50 |

Notes: 95% confidence intervals in brackets based on standard errors clustered at the country level. *p<0.1, **p<0.05, ***p<0.01. LMICs=low- and middle-income countries. HICs=high income countries. GOV=predominantly government-funded health system. SHI=predominantly social health insurance-funded health system. OOP=predominantly out-of-pocket-funded health system. HE=health expenditure. pc=per capita. FFS=fossil fuel subsidies. GDP=gross domestic product. NCD=non-communicable diseases. FE=fixed effects. IV=instrumental variable. Classification into predominantly government-, social health insurance-, or out-of-pocket-funded health systems available in Gabani et al.(2023)

Table A5. Impacts of petroleum product subsidies, instead of total FFS (coal, natural gas, petroleum products, and electricity), on total, domestic, and government health expenditure (IV results)

|  |  | **Total HE** | **Domestic HE** | **Government HE** |
| --- | --- | --- | --- | --- |
|  | Ln(FFS pc) | Ln(HE pc) | Ln(HE pc) | Ln(HE pc) |
| Ln(FFS pc) |  | -0.061*** | -0.063*** | -0.042 |
|  |  | [-0.103,-0.019] | [-0.105,-0.021] | [-0.097,0.014] |
| Ln(general government expenditure pc) | -1.120** | 0.004 | -0.001 | 0.054 |
|  | [-2.175,-0.064] | [-0.286,0.294] | [-0.300,0.298] | [-0.506,0.614] |
| Ln(CO2 emissions pc) | -0.950 | 0.035 | 0.074 | 0.392 |
|  | [-3.316,1.416] | [-0.239,0.309] | [-0.209,0.357] | [-0.139,0.924] |
| Ln(GDP pc) | 0.880* | 0.922*** | 0.929*** | 0.911*** |
|  | [-0.086,1.847] | [0.631,1.213] | [0.626,1.232] | [0.362,1.460] |
| Ln(Mortality from road traffic injury) | 1.492** | 0.071 | 0.087 | 0.270* |
|  | [0.332,2.652] | [-0.117,0.259] | [-0.094,0.267] | [-0.026,0.567] |
| Ln(Mortality from NCD) | 0.735 | 0.061 | -0.002 | -0.117 |
|  | [-4.158,5.628] | [-0.887,1.009] | [-0.922,0.918] | [-1.003,0.769] |
| Ln(Total energy imports*oil price) | 1.425*** |  |  |  |
|  | [0.746,2.104] |  |  |  |
| Trend | 0.033 | 0.001 | 0.002 | 0.006 |
|  | [-0.059,0.125] | [-0.017,0.020] | [-0.016,0.021] | [-0.021,0.033] |
| Country FE | Yes | Yes | Yes | Yes |
| Instrument strength test (first stage F-statistic) | F(1,66)=17.56*** |  |  |  |
| Durbin-Wu-Hausman endogeneity test |  | Chi-sq(1)=5.432** | Chi-sq(1)=5.634** | Chi-sq(1)=1.158 |
| # Observations | 299 | 299 | 299 | 299 |
| # Countries | 67 | 67 | 67 | 67 |

Notes: 95% confidence intervals in brackets based on standard errors clustered at the country level. *p<0.1, **p<0.05, ***p<0.01. HE=health expenditure. pc=per capita. FFS=fossil fuel subsidies. GDP=gross domestic product. NCD=non-communicable diseases. FE=fixed effects. IV=instrumental variable. The sample includes fewer countries that subsidize petroleum products, highlighted in grey in Table A2.

Table A6. Comparison between OLS and IV results with different sets of covariates

|  | **Domestic HE** | | | | | |
| --- | --- | --- | --- | --- | --- | --- |
|  | OLS | IV | OLS | IV | OLS | IV |
| Ln(FFS pc) | -0.011 | -0.083 | -0.004 | -0.051*** | -0.003 | -0.052*** |
|  | [-0.035,0.014] | [-0.190,0.024] | [-0.014,0.006] | [-0.082,-0.021] | [-0.013,0.008] | [-0.084,-0.020] |
| Ln(general government expenditure pc) |  |  |  |  | 0.102 | 0.057 |
|  |  |  |  |  | [-0.094,0.298] | [-0.137,0.250] |
| Ln(CO2 emissions pc) |  |  | -0.004 | 0.024 | -0.026 | 0.013 |
|  |  |  | [-0.145,0.138] | [-0.137,0.185] | [-0.170,0.118] | [-0.145,0.170] |
| Ln(GDP pc) |  |  | 0.935*** | 0.928*** | 0.833*** | 0.871*** |
|  |  |  | [0.916,0.955] | [0.906,0.950] | [0.631,1.034] | [0.673,1.069] |
| Ln(Mortality from road traffic injury) |  |  | 0.016 | 0.038 | 0.017 | 0.040 |
|  |  |  | [-0.096,0.129] | [-0.080,0.156] | [-0.094,0.127] | [-0.078,0.157] |
| Ln(Mortality from NCD) |  |  |  |  | 0.057 | 0.001 |
|  |  |  |  |  | [-0.511,0.624] | [-0.605,0.606] |
| Trend | 0.022*** | 0.032*** | -0.001 | 0.006 | 0.001 | 0.007 |
|  | [0.009,0.035] | [0.008,0.056] | [-0.009,0.007] | [-0.002,0.014] | [-0.012,0.013] | [-0.006,0.020] |
| Country FE | Yes | Yes | Yes | Yes | Yes | Yes |
| Instrument strength test (first stage F-statistic) |  | F(1,119)=35.57*** |  | F(1,119)=34.90*** |  | F(1,119)=36.29*** |
| Durbin-Wu-Hausman endogeneity test |  | Chi-sq(1)=2.770* |  | Chi-sq(1)=13.116*** |  | Chi-sq(1)=12.031*** |
| # Observations | 567 | 567 | 567 | 567 | 567 | 567 |
| # Countries | 120 | 120 | 120 | 120 | 120 | 120 |

Notes: 95% confidence intervals in brackets based on standard errors clustered at the country level. *p<0.1, **p<0.05, ***p<0.01. OLS=ordinary least squares. IV=instrumental variable. pc=per capita. FFS=fossil fuel subsidies. GDP=gross domestic product. NCD=non-communicable diseases. FE=fixed effects.

Table A7. Robustness checks: alternative and additional covariates

|  | **Total HE** | **Domestic HE** | **Government HE** |
| --- | --- | --- | --- |
|  | Ln(HE pc) | Ln(HE pc) | Ln(HE pc) |
| Baseline | -0.045*** | -0.052*** | -0.038* |
| *N=567, n=120* | [-0.076,-0.014] | [-0.084,-0.020] | [-0.080,0.003] |
| Replacing Ln(general government expenditure pc) with Ln(government revenues + borrowing pc) | -0.041** | -0.046** | -0.018 |
| *N=432, n=93* | [-0.076,-0.007] | [-0.082,-0.010] | [-0.079,0.043] |
| Adding indicators for Democracy/Dictatorship & Left/Right/Other regime | -0.045*** | -0.052*** | -0.038* |
| *N=562, n=119* | [-0.077,-0.013] | [-0.085,-0.019] | [-0.080,0.005] |
| Adding % population <15 & 65+ | -0.045*** | -0.052*** | -0.038* |
| *N=567, n=120* | [-0.076,-0.014] | [-0.084,-0.021] | [-0.080,0.004] |

Notes: 95% confidence intervals in brackets based on standard errors clustered at the country level. *p<0.1, **p<0.05, ***p<0.01. HE=health expenditure. pc=per capita. Government revenues and borrowing, and population age structure available from the World Bank’s World Development Indicators, databank.worldbank.org/source/world-development-indicators. Democracy and regime political orientation data available from Herre(2023).

Table A8. Robustness checks: alternative IVs and trend specifications

|  | **Total HE** | **Domestic HE** | **Government HE** |
| --- | --- | --- | --- |
|  | Ln(HE pc) | Ln(HE pc) | Ln(HE pc) |
| IV: Ln(Total energy imports, 2010-14 average*oil price) | -0.045*** | -0.052*** | -0.038* |
| Trend: linear | [-0.076,-0.014] | [-0.084,-0.020] | [-0.080,0.003] |
| IV: Ln(Total energy imports, 2010-14 average*oil price) | -0.046*** | -0.053*** | -0.039* |
| Trend: region-specific linear trends | [-0.077,-0.015] | [-0.085,-0.021] | [-0.081,0.003] |
| IV: Ln(Total energy imports, 2010-14 average*oil price) | -0.045*** | -0.052*** | -0.040** |
| Trend: quadratic | [-0.074,-0.017] | [-0.081,-0.022] | [-0.078,-0.002] |
| IV: Ln(Total energy imports, 2010-14 average*oil price) | n.a. | n.a. | n.a. |
| Trend: year fixed effects |  |  |  |
| IV: Ln(Total energy imports, current*oil price) | -0.053 | -0.059 | -0.062 |
| Trend: linear | [-0.123,0.017] | [-0.130,0.012] | [-0.208,0.084] |
| IV: Ln(Total energy imports, current*oil price) | -0.057 | -0.065* | -0.069 |
| Trend: region-specific linear trends | [-0.128,0.013] | [-0.137,0.008] | [-0.215,0.077] |
| IV: Ln(Total energy imports, current*oil price) | -0.054 | -0.059 | -0.063 |
| Trend: quadratic | [-0.124,0.017] | [-0.131,0.013] | [-0.211,0.084] |
| IV: Ln(Total energy imports, current*oil price) | -0.071 | -0.075 | -0.112 |
| Trend: year fixed effects | [-0.264,0.122] | [-0.270,0.120] | [-0.534,0.310] |
| IV: Ln(average FFS in the region) | -0.052* | -0.059** | -0.032 |
| Trend: linear | [-0.106,0.001] | [-0.114,-0.003] | [-0.119,0.056] |
| IV: Ln(average FFS in the region) | -0.060** | -0.069** | -0.050 |
| Trend: region-specific linear trends | [-0.111,-0.010] | [-0.122,-0.017] | [-0.121,0.022] |
| IV: Ln(average FFS in the region) | -0.053** | -0.058** | -0.034 |
| Trend: quadratic | [-0.105,-0.001] | [-0.112,-0.005] | [-0.117,0.049] |
| IV: Ln(average FFS in the region) | -0.064 | -0.068 | -0.028 |
| Trend: year fixed effects | [-0.175,0.047] | [-0.183,0.046] | [-0.224,0.168] |

Notes: 95% confidence intervals in brackets based on standard errors clustered at the country level. *p<0.1, **p<0.05, ***p<0.01. HE=health expenditure. pc=per capita. FFS=fossil fuel subsidies. IV=instrumental variable. Top panel: IV is the natural logarithm of the product between the international crude oil price and countries’ total annual energy imports averaged across 2010-14; year fixed effects would be collinear with the international crude oil price, and thus the IV, as average energy imports are constant over time. Middle panel: IV is the natural logarithm of the product between the international crude oil price and countries’ current total annual energy imports. Bottom panel: IV is the natural logarithm of FFS per capita across the other countries in the region (i.e., leave-one-out average).

Table A9. Robustness check: winsorization

|  | **Total HE** | **Domestic HE** | **Government HE** |
| --- | --- | --- | --- |
|  | Ln(HE pc) | Ln(HE pc) | Ln(HE pc) |
| Baseline | -0.045*** | -0.052*** | -0.038* |
|  | [-0.076,-0.014] | [-0.084,-0.020] | [-0.080,0.003] |
| Winsorization at top and bottom 1% | -0.048*** | -0.055*** | -0.041* |
|  | [-0.080,-0.016] | [-0.088,-0.022] | [-0.083,0.002] |
| Winsorization at top and bottom 5% | -0.049** | -0.053** | -0.039 |
|  | [-0.094,-0.005] | [-0.098,-0.008] | [-0.102,0.023] |

Notes: 95% confidence intervals in brackets based on standard errors clustered at the country level. *p<0.1, **p<0.05, ***p<0.01. HE=health expenditure. pc=per capita. Winsorization is a procedure to attenuate outliers by setting values below the 1^st^ and above the 99^th^ percentiles to the 1^st^ and 99^th^ percentile values (or at any other point of the distribution). Winsorization was performed for all variables, including outcomes, FFS, the IV, and covariates.
